# Analytical Sketch of Cancer of the Stomach, and of Its Relations to Chronic Gastritis and Gastralgia

**Published:** 1844-07

**Authors:** 


### ART. XVII.

*Précis Analytique sur le Cancer de l'Estomac et sur ses Rapports avec la Gastrite Chronique et les Gastralgies.* Par le Docteur BARRAS. —*Paris*, 1842.

*Analytical Sketch of Cancer of the Stomach, and of its Relations to Chronic Gastritis and Gastralgia.* By Dr. BARRAS.—*Paris*, 1842. 8vo, pp. 134.

THE name of M. Barras has been long connected with the class of diseases to which the present small work refers, from his having published many years ago, a well known work upon Gastralgia. The particular aspect under which he considers the subject of gastric diseases in the pages before us is one of extreme interest, and his observations are evidently those of an observer who has studied the diseases he proposes to treat of.

The remarks made by the author on the morbid anatomy of cancerous disease of the stomach simply show that he accepts the current doctrines respecting the physical characters of that affection. The anatomical conditions of the diseased organ could not supply him with any direct aid in the immediate object of his work, that is, to establish the points serving, clinically, to distinguish cancerous, inflammatory, and nervous affections of the stomach. He observes, however, that few cases have ever been met with in which cancerous disease affected the entire organ, and comments upon the disproportionate frequency with which its orifices and lesser curvature suffer. On the other hand, gastric inflammation spreads over a wider surface than cancer, but a much more limited one than gastralgia. The neurosis may possibly originate in a single point, but, unless controlled at the outset, it gradually extends, implicates the entire organ, and even spreads to the liver and intestines.

M. Barras' experience (and that of every practical physician will here agree with his,) is, that cancer of the stomach is infinitely rarer than gastralgia. Within the last eighteen years he has observed at least five hundred cases of gastralgia, and not more than thirty of cancer. He considers chronic primary gastritis as rare as cancer, if not actually more so. We believe him here, too, to be correct; but he makes an almost ludicrous error (at the present day it can scarcely be styled otherwise,) in including the condition of the organ described by M. Louis as "softening with attenuation of the mucous membrane," among the number of its inflammations. Even M. Louis himself now knows that he mistook the effects of the gastric juice for a pathological condition.

Gastralgia M. Barras considers somewhat more common in females than males: cancer, on the contrary, he believes to occur much more frequently in men than women. This latter belief is certainly warranted by his own experience, for it appears that, of thirty persons in whom he ascertained the existence of the disease, twenty-six were men and four only women. It is obvious that could these numbers be regarded as significative of general experience, they would furnish an important element of diagnosis, but we have no hesitation in affirming that such is not the fact. The number of cases on which it rests is clearly insufficient to give weight to the statement.

Neurosis of the stomach is commonly met with in persons aged between fifteen and forty-five. Unlike M. Barras, we have, however, met with the affection before puberty; nor are we so certain of its never originating later than the forty-fifth year. Cancer of the stomach is principally observed between the thirtieth and sixtieth years of life. Chronic gastritis attacks both sexes indifferently, and, according to the author, is met with at all ages,—a proposition we are neither enabled to affirm nor deny.

Both gastralgia and cancer of the stomach are hereditary affections, according to M. Barras' opinion; and next to hereditary influence he considers "a constitution impaired by scrofulous, syphilitic, arthritic, rheumatismal, dartrous, or other affections implicating the economy generally," its most powerful cause. This latter proposition, if it mean anything, means simply that people of bad constitution have a stronger tendency than others to cancer of the stomach. Even thus understood, the point is mere matter of opinion, at least demonstrative evidences of its justness has never been tendered by any writer. But hear the author:

"A circumstance *proving* the affinity of scrofula and cancer is, that tubercles of the lungs,—productions which though not perfectly identical with, are yet allied to *scirrhous tubercles*,—sometimes form in young persons born of parents cut off by cancer. Thus, two children and three grandchildren of a lady who died of uterine cancer became phthisical, and no cause could be assigned for the disease except the cancerous affection of their parent."

This is so utterly absurd, that we should certainly not have transferred the passage to our pages, but that the medical press teems with works in which etiological questions of the most serious character are settled in a similar spirit, and that we desire to use the opportunity of warning our readers against such illogical platitudes: *hæc tu Romane caveto.* Labours of another kind, and mental qualifications of another order from those undertaken by, or possessed by M. Barras, are required for the settlement of these high problems of pathology.

Assuming that cancer of the stomach is an hereditary disease, (we cannot help, as well as the writer, regarding it as such, though this proposition, too, is unproved,) he points to the non-hereditary character of gastralgia as constituting a ground of distinction between the affections.

When a disease of the stomach, says M. Barras, has been produced by immoderate hemorrhage, venesection carried to extremes, the abuse of other antiphlogistic remedies, and particularly of mucilaginous drinks, fasting, low diet, excessive coition, masturbation, &c., we may almost feel positive, that it is nervous in character. Nevertheless, these causes of debility, impoverishing, as they do, the blood, creating a proportional preponderance on the part of the lymphatic system, and so a kind of semi-scrofulous condition of the system, might, in the opinion of this particularly rigid reasoner, give rise to a predisposition to scirrhus. All this, we need scarcely say, is pure matter of hypothesis.

The writer refers to some cases of cancer of the stomach in which the causation of the disease was, according to him, evident. Two officers of rank, desirous of taking an active part in a campaign about to be entered upon, found that the ease of a long period of inaction had given such lusty proportions to their figures,—they had, in a word, grown so enormously fat, that the idea of their campaigning seemed an absurdity. They drank vinegar in abundance, thinned themselves down to respectable dimensions, and died of cancer of the stomach "in consequence."

At least so says M. Barras; but in the one case no post-mortem examination was instituted, and the other he only heard of from a brother officer of the victim of vinegar! Further, adds the writer, it is stated by M. de Rienzi, that the inhabitants of the Haonai islands being very commonly incommoded by unseemly accumulations of fat, drink *kava*, (the Haonai vinegar,) which thins them to the perfection of their desires; but the kava-drinkers soon suffer in their stomachs, and die in a state of marasmus. M. Barras thinks that they "probably die of cancer of that organ,"—a conclusion less judicious, perhaps, than convenient for his purpose.

Such being the points of distinction of the three affections connected with their presumed etiology, their comparative semeiology may be inquired into.

Cancer of the stomach is always an idiopathic malady; on the contrary, chronic gastritis is sometimes sympathetic of latent inflammation of the brain or its membranes; and gastralgia frequently thus dependent upon disease of the brain, medulla spinalis, the ganglia or plexuses of the abdominal nerves, the kidneys, bladder, or uterus.

Gastralgia and chronic gastritis, no matter how slight they may be, never run a completely latent course; scirrhus of the stomach may, on the contrary, exist for a length of time with all outward appearances of good health, and without local pain or disturbance of digestion.

The early symptoms of cancer of the stomach are well sketched in the following passage:

"Pale tongue, or of natural colour; mouth clammy, and with mawkish or sometimes a bitter or acid taste; failure of appetite; laborious digestion, especially of solids; discomfort, uneasiness, and sensation of weight almost habitually in the region of the stomach, or dull and deep-seated pains in this region, increasing under pressure, felt when the stomach is empty, but most severe immediately after the ingestion of food; breath heavy and nauseous; eructations with disagreeable sour and caustic taste; great quantities of flatus. At a later period the epigastric pain is sometimes lancinating with occasional exacerbations, and gradually becomes continuous; the bowels grow more and more obstinately constipated, and nausea with slight (and at first rare) vomiting of watery, ropy, viscid, and glairy, sour or insipid matters occurs. Still later, a few mouthfuls of food are rejected after meals. The colour commences to change, and becomes pale, wan, and sallow."

Upon this alteration of colour of the skin, which gradually passes into the almost pathognomonic straw-coloured tint of cancer, the author lays very considerable stress;—and deservedly. We for our own part, believe firmly,—we have experienced the difficulty, and witnessed others labouring under it also,—that without it, the disease can scarcely, in no small number of cases, be distinguished satisfactorily from chronic gastritis. We of course mean so long as it has not advanced further than the stage indicated by the symptoms just enumerated. Yet there are, in well marked instances of chronic gastritis, occasionally observable a group of conditions extremely characteristic of this affection. Thus, the tongue is red, dry, and pinched; the patient suffers from thirst and sensation of heat in the stomach; the lower part of the thorax feels as if bound down firmly by a weight or ligature; the pain felt at the epigastrium is rather inconvenient than severe, and is scarcely ever present when the stomach is empty; vomitings occur at irregular periods, before or after meals; colic pains, and diarrhea supervene if, as is very frequently the fact, the inflammation spread to the intestine, slight fever with evening exacerbation sets in, and the lips, conjunctivæ, and cheeks acquire a violet tint, which is more marked during the progress of digestion, and of the febrile paroxysms than at other times.

"In gastralgia the appetite is natural, impaired, or increased, perverted, depraved, capricious, fantastic, irregular ; liquids are digested with greater difficulty than solids, whereas, the reverse is the case in cancer; the digestive process is sometimes easy, and is always affected in the end, in spite of the discomfort and suffering, which it frequently produces; the breath is free from bad smell, eructation of air free from disagreeable taste occurs frequently ; pain at the epigastrium, often of greater severity than in cancerous disease, occurring in irregular paroxysms, shooting to the shoulders, and the walls of the chest, and diminishing instead of increasing under pressure, and on the ingestion of food; besides this there are curious indescribable sensations felt at the epigastrium, and singular pulsations."

It is to be remembered that pain may sometimes be altogether absent in gastralgia for a time—almost a contradiction in words. The description of neurosis of the stomach, which may be most readily confounded with cancer of the organ, is that principally characterized by vomiting of the ingesta. But in the gastralgic affection, the colour of the patient's skin undergoes no unhealthy change, and his strength and flesh do not give way.

The writer dwells emphatically upon the fact that cancer of the stomach is not attended with hypochondria. His own experience, coupled with the circumstance, that, of forty cases of cancer related in Chardel's treatise, not one furnishes an instance of hypochondriacal complication, fully justifies his statements on this point. It is, we perfectly agree with M. Barras, the nervous affection of the abdominal viscera which is allied with that distressing condition; insomuch that its existence will aid materially in excluding the idea of cancer from our diagnosis.

Chronic gastritis and gastralgia affect organs, more or less distant from that specially implicated, by *sympathy;* cancer does so more rarely, and by *absorption.*

In the second period of cancerous disease, the symptoms of the first become more marked; nausea and vomiting, above all, grow more frequent, especially when the disease is seated at the pylorus, and the solids vomited are sometimes mixed with, or followed by, blackish matters, one of the almost pathognomonic signs of the affection. Emaciation, debility, and languor increase rapidly; the colour becomes truly cancerous, as already referred to. Gurgling of fluids may now be detected by pressing the epigastrium, and local tension or an actual tumour discovered there by manual examination and percussion. The patient ceases to enjoy any intervals of ease,—his hours wear slowly on in unceasing suffering; nevertheless, the moral condition is so opposed to hypochondria, that sometimes patients have been known, up to the very day of death, to dwell on the chances of recovery. It is just, however, to add that the cancerous sufferer is generally morose, sour, and anxious about himself, but still he is not, correctly speaking, hypochondriacal. The pulse is feeble and slow, the skin cold rather than hot, in a word, the power of reaction to produce fever, seems wanting.

Nevertheless, during this stage of the disease, the only truly pathognomonic sign of its existence, is the discovery of a tumour at the epigastrium; and it is not until the third stage of the malady, at a period when it is hardly an exaggeration to call the patients moribund, that its diagnosis can, independently of the detection of tumour, be established beyond the reach of error. During the third stage, all strength is gone, the patient has frequent fainting-fits, his emaciation is carried to the extremest pitch; the features are decomposed, pinched, and cadaverous; the lower extremities frequently become anasarcous, the face bloated, the abdomen the seat of effusion; the frequency of vomiting increases, as likewise the quantity of matter vomited, and the patient expires, not very seldom, retaining his consciousness to the last, after having vomited an enormous quantity of the usual coffee-dreg looking matters.

M. Barras proceeds to the consideration of the irregularities occasionally presenting themselves in the course of cancer of the stomach. Among these are the extraordinary remissions sometimes observed, and of which we have ourselves met with two instances. Gastric cancer, as M. Barras says, may have advanced so far as to have produced black vomiting, and yet shall undergo such temporary suspension, that the outward appearances of health shall all for a time return. This is a most curious fact in pathology, but not more so than the equally well-established one, that phthisis will sometimes, (why or wherefore nobody knows, except that we know the fortunate occurrence is not the result of treatment,) stop short for a time in its progress. It will be well for cancerous persons to bear in mind that the most common cause of relapse of the symptoms in such cases as those referred to is excess at table. We regard, with M. Barras, the objection which may be urged to the view now taken of the nature of these cases; namely, that they are at first gastralgic, and subsequently become cancerous, as devoid of any degree of force. Gastralgia does *not* pass into cancer.

But, unfortunately for the facility of diagnosis, the three affections we have been considering may become associated. It is not very uncommon for chronic gastritis or gastralgia, to coexist with cancer of the stomach. Nothing but the deepest attention to the character and succession of the symptoms will in such cases detect the true nature of the malady. The reality of their occasional coexistence is proved by post-mortem examinations; as well as the reality of such a state as perfectly uncomplicated cancer.

M. Barras' observations on the treatment of cancer of the stomach are sufficiently judicious, but contain nothing positively new. He has particular confidence in the use of Vichy waters, with extract of conium taken internally, and also applied externally in the form of plaster. He correctly insists upon the importance of extreme attention to diet,—without this, in truth, medicine may be poured into the stomach without the remotest chance of benefiting the sufferer. Ioduret of potass, white oxide of antimony, *calomel*, acetate of potass, are the medicines which he thinks may be beneficially associated according, to circumstances, with the conium. Externally, carrot-poultices, mercurial plasters and frictions, or preparations of iodine or conium used in the same ways; local abstraction of blood, blisters, and baths; the application of emollients if there be evidence of inflammation in the neighbourhood of the scirrhous tumour, and narcotics internally if there be pain and signs of neurosis.

